# Treatment-naïve lung cancer presenting with spinal metastases: a national study of survival, surgery, and the role of predictive biomarkers

**DOI:** 10.1007/s00701-026-06914-3

**Published:** 2026-05-19

**Authors:** Martin Isaksson, Per Andersson, Helena Nyström, Catharina Parai, Pedram Tabatabaei, Olof Westin, Björn Knutsson, Johan Wänman

**Affiliations:** 1 Department of Diagnostics and Intervention, Umeå University, Umeå, Sweden; 2 Department of Clinical Sciences, Umeå University, Umeå, Sweden; 3 Department of Orthopaedics, Clinical Sciences, University of Gothenburg, Gothenburg, Sweden

**Keywords:** Biomarkers, Lung cancer, Spinal metastases, Postoperative survival, Spinal metastases

## Abstract

**Purpose:**

Spinal metastasis as the first manifestation of lung cancer, presents challenges in diagnosis, prognosis, and treatment planning. This study aims to evaluate postoperative survival outcomes and to identify prognostic factors in treatment-naïve patients undergoing surgery for spinal metastases, focusing on clinical and molecular variables.

**Methods:**

This retrospective cohort study included 149 patients who underwent surgery for spinal metastases from lung cancer between 2011 and 2023. Data were obtained from the Swedish National Register for Spine Surgery (Swespine) and the Swedish National Lung Cancer Register (SNLCR). Patients were grouped based on whether spinal metastasis was the initial manifestation of lung cancer or occurred during known disease. Kaplan–Meier analysis and Cox proportional hazards models were used to assess postoperative survival and prognostic factors.

**Results:**

Of the 149 patients, 114 (77%) presented with spinal metastasis as the initial sign of lung cancer. Median survival in this group was 6 months, compared to 3 months in patients with a prior confirmed lung cancer diagnosis (*p* = 0.022). In the initial presentation group, longer survival after surgery was observed in patients with predictive biomarkers (8 vs. 5 months, *p* = 0.003), preserved ambulatory function (8 vs. 3 months, *p* = 0.002), and better WHO performance status (*p* < 0.001). In multivariable analysis, biomarker status, WHO performance status, ambulatory function, and age were independently associated with post-operative survival.

**Conclusion:**

Patients undergoing surgery for spinal metastases as the initial manifestation of lung cancer form a distinct subgroup. Early functional and molecular assessment may improve patient selection and surgical outcome in this population.

**Supplementary Information:**

The online version contains supplementary material available at 10.1007/s00701-026-06914-3.

## Introduction

Lung cancer is the leading cause of cancer-related mortality worldwide, accounting for approximately 2.2 million new cases and 1.8 million deaths annually [19]. A considerable number of patients are diagnosed at an advanced stage, with up to 40% developing bone metastases, most often in the spine. Lung cancer is the third most frequent primary malignancy to metastasize to bone, following prostate and breast cancer [28].

Metastatic spinal cord compression (MSCC) is one of the most severe complications to spinal metastasis, which can result in neurological deficits, including paraplegia, tetraplegia, and incontinence [5, 8]. Prognosis in patients with lung cancer and spinal metastasis has traditionally been poor, particularly in patients with MSCC, with a reported median survival of 2–4 months [12, 24, 26].

The introduction of targeted therapies and immune checkpoint inhibitors has substantially improved survival outcomes in selected subgroups of non-small cell lung cancer (NSCLC), which accounts for approximately 85% of lung cancer cases [10]. NSCLC includes histological subtypes such as adenocarcinoma, squamous cell carcinoma, and large cell carcinoma [18]. Molecular profiling has identified actionable mutations, such as epidermal growth factor receptor (EGFR) and anaplastic lymphoma kinase (ALK) for which targeted treatments are now available. Additionally, Programmed Death Ligand 1 (PD-L1) expression can predict response to immunotherapy [9, 18]. These treatments have altered the prognosis and may influence the indication and timing for surgical interventions in metastatic disease [1, 3, 6, 16, 27]. Despite advances in oncological and surgical care, the optimal management of painful skeletal metastases remains without clear consensus, necessitating individualized multimodal strategies given the diverse treatment goals and biological behavior of metastases at different anatomical sites [13].

In a subset of patients, spinal metastasis is the first clinical manifestation of previously undiagnosed lung cancer [4, 25]. These cases present distinct diagnostic and therapeutic challenges, as the primary tumour is unknown at presentation, key information, such as molecular profile and disease stage is unavailable at the time of surgical decision-making. Existing studies have largely focused on patients with established lung cancer diagnoses, leaving a gap in knowledge regarding outcomes in treatment-naïve patients who undergo surgery before full oncological workup [1, 3, 6, 16, 27]. This study aimed to investigate postoperative survival in this specific group, focusing on the prognostic impact of histological subtype, predictive biomarkers, and clinical status, using a large national cohort with linked surgical and oncological data.

## Methods

### Study design, data sources, and setting

This retrospective multi-register cohort study used data from two national quality registers in Sweden: The Swedish National Register for Spine Surgery (Swespine) and the Swedish National Lung Cancer Register (SNLCR). Swespine operational since 1993, started collecting data on surgeries for spinal metastases in 2006. As of 2023, 47 clinics, representing approximately 95% of all spine surgery clinics nationwide. Swespine collects data on approximately 85% of all surgeries nationwide, achieving a one-year follow-up completion rate of approximately 70%. Established in 2002, the SNLCR covers more than 95% of all lung cancer cases in Sweden. Designed to support quality assurance and research, it contains data on patient demographics, diagnostic procedures, histopathology, staging, and primary treatment.

### Patient selection

Patients undergoing surgery for spinal metastases from lung cancer or cancer of unknown primary were identified through Swespine between March 17, 2011, and December 31, 2023.

**Fig. 1:**
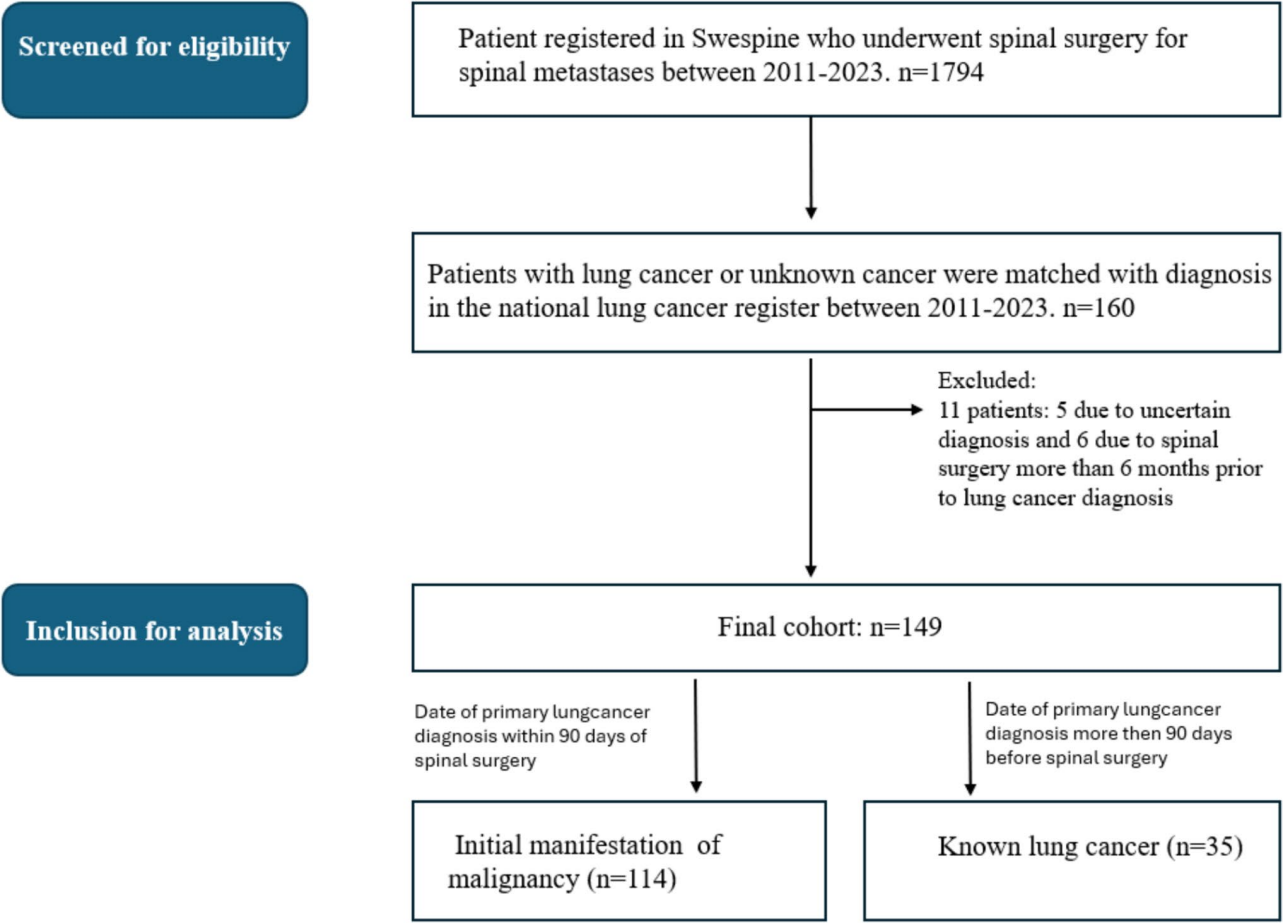
Flow chart. Flow chart showing how patients who underwent surgery for spinal metastasis registered in Swespine (2011–2023) were screened for eligibility, then matched with lung cancer diagnosis in the national lung cancer register. The cohort were then categorized into two groups based on cancer status at the time of spinal surgery: those with spinal metastases as the initial manifestation of malignancy (IMM), defined by a lung cancer diagnosis made within 90 days postoperatively, and those with previously known lung cancer

These entries were linked to the SNLCR using the Swedish unique personal identity number to obtain additional cancer-related data. From the initial 160 patients identified, 11 were excluded: five due to an uncertain diagnosis and six due to spinal surgery being performed more than six months prior to lung cancer diagnosis. The final study population comprised 149 patients (Fig. 1, Flow chart).

### Included variables

From the SNLCR we extracted age at diagnosis, sex, smoking status, TNM stage, presence of predictive biomarkers (EGFR, ALK, PD-L1, ROS1, BRAF, RET), WHO performance status, date of diagnosis, and primary cancer-treatment type. From Swespine we collected age at the time of spinal surgery, date of surgery, pre-operative neurological function according to the Frankel classification (ambulatory [Frankel D–E] or non-ambulatory [Frankel A–C]), indication for surgery, type of surgical intervention and anatomical location of spinal metastases (cervical, thoracic, or lumbar) and quality-of-life outcomes, including EQ-5D, 6 weeks after surgery.

#### Statistical analysis

Continuous variables were presented as medians with interquartile ranges (IQR), and categorical variables as counts and percentages. Group comparisons of proportions were conducted using the Chi-square test.

Patients were stratified into two groups based on cancer status at surgery: those with spinal metastases as the initial manifestation of malignancy (IMM) and those with previously known lung cancer. Patients were categorized as having spinal metastases as the IMM if the primary tumour was unknown at the time of surgery and subsequently diagnosed within 90 days post-surgery. The 90-day period was selected to account for the diagnostic process typically initiated by spine surgeons, including postoperative imaging, histopathology, and molecular profiling. As lung cancer in these cases often are confirmed after referral to oncologists or pulmonologists, who register patients in the national lung cancer registry, this timeframe accommodates delays inherent in interdepartmental workflows, biopsy processing, and referral procedures (see flow chart, Fig. 1). A sensitivity analysis for this criterion was performed using stratifications of 30, 60, and 90 days between the primary diagnosis and the spinal surgery. Kaplan–Meier survival analysis was used to estimate postoperative survival. A Cox proportional hazards regression model was applied to assess associations between patient variables and postoperative survival in both the entire cohort and the subgroup with unknown primary tumours. The proportional hazards assumption was evaluated using Schoenfeld residuals for each covariate as well as for the model globally and no violations of the assumption were detected. Multicollinearity was assessed using variance inflation factors (VIF) and tolerance statistics derived from a linear regression model including all candidate predictors. All variables demonstrated VIF values < 2 and tolerance values > 0.80, indicating no evidence of multicollinearity. The associations are presented as hazard ratios (HRs) with 95% confidence intervals (CIs). The cox regression analyses were performed on both raw and imputed datasets. Multiple imputation of missing data in the variables ‘ambulation at the time of surgery’ and ‘WHO performance status’ was conducted to allow for robust multivariable analyses. The statistical analyses were performed using IBM SPSS Statistics version 29.0 (IBM Corp., Armonk, NY, USA) or R version 4.3.0 (R Foundation for Statistical Computing, Vienna, Austria). A *p*-value < 0.05 was considered statistically significant.

## Results

### Patient cohort and clinical characteristics

Patient characteristics at the time of primary lung cancer diagnosis are presented in Table 1, and at the time of spinal surgery in Table 2. The final cohort included 149 patients, of whom 114 (77%) presented with spinal metastases as the initial manifestation of lung cancer and 35 patients (23%) with a previously known lung cancer diagnosis at the time of surgery.

**Table 1:** Clinical characteristics at the primary diagnosis of lung cancer

	Spinal surgery as initial manifestation of lung cancer, *n* (%)	Spinal surgery; Known lung cancer, n (%)	Total	*P*-value
Number of patients	114	35	149	
Type of tumor				0.178
Squamous cell lung cancer	11 (9,6)	8 (22,9)	19	
Small cell	15 (13,2)	2 (5,7)	17	
adenocarcinoma	74 (64,9)	21 (60)	95	
Large cell/poorly differentiated non-small cell carcinoma	11 (9,6)	2 (5,7)	13	
Others*	3 (2,6)	2 (5,7)	5	
Age at diagnosis (Median IQR)	68 (62–74)	67 (54–72)	68 (61–74)	0.226
Sex				0.674
Male	60 (52,6)	17 (48,6)	77	
Female	54 (47,4)	18 (51,4)	72	
Smoking				0.834
Yes	93 (81,6)	28 (80)	121	
No	21 (18,4)	7 (20)	28	
TNM-Stadium				0.086
*T-stadium*				
T1	25 (21,9)	8 (22,9)	33	
T2	28 (24,6)	4 (11,4)	32	
T3	18 (15,8)	10 (28,6)	28	
T4	39 (34,2)	13 (37,1)	52	
Tx	4 (3,5)	0 (0)	4	
*N-Stadium*				0.636
N0	35 (30,7)	13 (37,1)	48	
N1	11 (9,6)	2 (5,7)	13	
N2	40 (35,1)	9 (25,7)	49	
N3	25 (21,9)	9 (25,7)	34	
Nx	3 (2,6)	2 (5,7)	5	
*M-stadium*				< 0.001
M0	2 (1,8)	17 (48,6)	19	
M1	112 (98,2)	18 (51,4)	130	
EFGR				0.897
positive	18 (26,1)	4 (23,5)	22	
Negative	48 (69,6)	12 (70,6)	60	
inconclusive	3 (4,3)	1 (5,9)	4	
PDL1				0.922
Positive	21 (53,8)	5 (55,6)	26	
Negative	16 (41,0)	4 (44,4)	20	
inconclusive	2 (5,1)	0 (0)	2	
PDL1-grade				0.087
≥ 1%	6 (28,6)	1 (20)	7	
≥ 20%	3 (14,3)	3 (60)	6	
≥ 50%	12 (57,1)	1 (20)	13	
ALK				0.874
Positive	5 (10)	1 (9,1)	6	
Negative	43 (84,3)	10 (90,9)	53	
inconclusive	3 (5,9)	0 (0)	3	
ROS1				0.182
Positive	0 (0)	1 (12,5)	1	
Negative	30 (90,9)	7 (87,5)	37	
inconclusive	3 (9,1)	0 (0)	3	
Braf				0.610
Positive	0 (0)	0 (0)	0	
Negative	19 (95)	5 (100)	24	
inconclusive	1 (5)	0 (0)	1	
Ret				No statistics
Positive	0 (0)	0 (0)	0	
Negative	9 (90)	0 (0)	9	
inconclusive	1 (10)	0 (0)	1	
WHO performance status				< 0.001
0	10 (8,8)	13 (37,1)	23	
1	36 (31,6)	19 (54,2)	55	
2	26 (22,8)	2 (5,7)	28	
3	29 (25,4)	1 (2,9)	30	
4	6 (5,3)	0 (0)	6	
Missing	7 (6,1)	0 (0)	7	
Primary treatment of lung cancer				< 0.001
Pulmonary surgery	3 (26,3)	7 (20)	10	
Curative intent chemoradiotherapy	2 (1,8)	4 (11,4)	6	
Radiotherapy	6 (5,3)	0 (0)	6	
Pharmacological treatment	41 (36,0)	18 (51,4)	59	
Other tumor treatment	11 (9,6)	0 (0)	11	
Radiotherapy against distant metastasis	24 (21,1)	2 (5,7)	26	
Stereotactic radiotherapy	0 (0)	2 (5,7)	2	
Missing	27 (23,7)	2 (5,7)	29	

*Others include adenosquamous carcinoma (*n* = 1), pleomorphic/sarcomatoid (*n* = 1), and unclassified (*n* = 3)

**Table 2:** Clinical characteristics at the time of spinal surgery

	Spinal surgery; IMM* lung cancer, n (%)	Spinal surgery; Known lung cancer, n (%)	Total	*P*-value
Indication of surgery				0.690
Loss of neurological function	43 (37,7)	9 (25,7)	52	
Pain	11 (9,6)	3 (8,6)	14	
Progressive deformity	0 (0)	0 (0)	0	
Loss of neurological function, and pain	33 (28,9)	13 (37,1)	46	
Loss of neurological function and progressive deformity	4 (3,5)	1 (2,9)	5	
Pain and progressive deformity	3 (2,6)	0 (0)	3	
Loss of neurological function, pain and progressive deformity	4 (3,5)	3 (8,6)	7	
Missing	16 (14,0)	6 (17,1)	22	
Frankel				1.00
A	3 (2,6)	1 (2,9)	4	
B	4 (3,5)	1 (2,9)	5	
C	36 (31,6)	11 (31,4)	47	
D	39 (34,2)	11 (31,4)	50	
E	14 (12,3)	4 (11,4)	18	
Missing	18 (15,8)	7 (20)	25	
Ambulation at the time of surgery				0.878
Yes	53 (46,5)	15 (42,9)	96	
No	43 (37,7)	13 (37,1)	28	
Missing	18 (15,8)	7 (20)	25	
Location of spinal lesion				0.372
Cervical	3 (2,6)	1 (2,9)	4	
Thoracic	66 (57,9)	23 (65,7)	89	
Lumbar	17 (14,9)	4 (11,4)	21	
Sacral	1 (0,9)	2 (5,7)	3	
Missing	27 (23,7)	5 (14,3)	32	
Reported as unknown tumour at the time of spinal surgery				< 0.001
yes	72 (63,2)	2 (5,7)	74	
No	42 (36,8)	33 (94,3)	75	
Tumour resection				0.193
Yes	95 (83,3)	27 (77,1)	122	
No	15 (13,2)	8 (22,9)	23	
Missing	4 (3,5)	0 (0)	4	
Surgical procedure				
Posterior decompression and stabilization	84 (73,7)	23 (65,7)	107	
Anterior decompression and stabilization	1 (0,9)	0 (0)	1	
Combined approach (Anterior and posterior decompression and stabilization)	5 (4,4)	0 (0)	5	
Decompression only	16 (14,0)	7 (20)	23	
Stabilization only	3 (2,6)	1 (2,9)	4	
Missing	5 (4,4)	4 (11,4)	9	

*IMM: Spinal surgery as initial manifestation of malignancy of lung cancer defined as less than 90 days between spinal surgery and primary diagnosis of lung cancer

The median age at both diagnosis and surgery was 68 years (IQR 61–74). The most common histological subtype was adenocarcinoma (*n* = 95), followed by squamous cell carcinoma (*n* = 19), small cell carcinoma (*n* = 17), large cell/poorly differentiated NSCLC (*n* = 13), and other/unclassified histology (*n* = 5). Thoracic spinal metastases (*n* = 89, 60%) was the most common location. In total, 68 patients were ambulatory before spinal surgery (Frankel D and E) and 56 were non-ambulatory (Frankel A, B, C). Data Frankel classification at the time of surgery was missing for 25 patients (Table 2).

### Primary treatment of lung cancer

Systemic therapy was the most common primary treatment (*n* = 59), followed by radiotherapy targeting distant metastases (*n* = 26), surgery (*n* = 10), curative-intended chemoradiotherapy (*n* = 6), radiotherapy (*n* = 6), stereotactic radiotherapy (*n* = 2), and other tumour treatments (*n* = 11). Data were missing in 29 cases (Table 1).

### Spinal surgical treatment

Loss of neurological function was the most common indication for surgery. Posterior decompression and stabilization was the most common surgical method (*n* = 107), followed by decompression only (*n* = 23) and combined approaches, with anterior and posterior decompression and stabilization (*n* = 5). One patient underwent anterior decompression and stabilization, four patients underwent stabilization only, and data were missing for nine patients. A total of 122 patients underwent tumour resection, with intralesional excicion being the most common procedure. For details on surgical procedures se Table 2.

### Survival outcomes

**Fig. 2:**
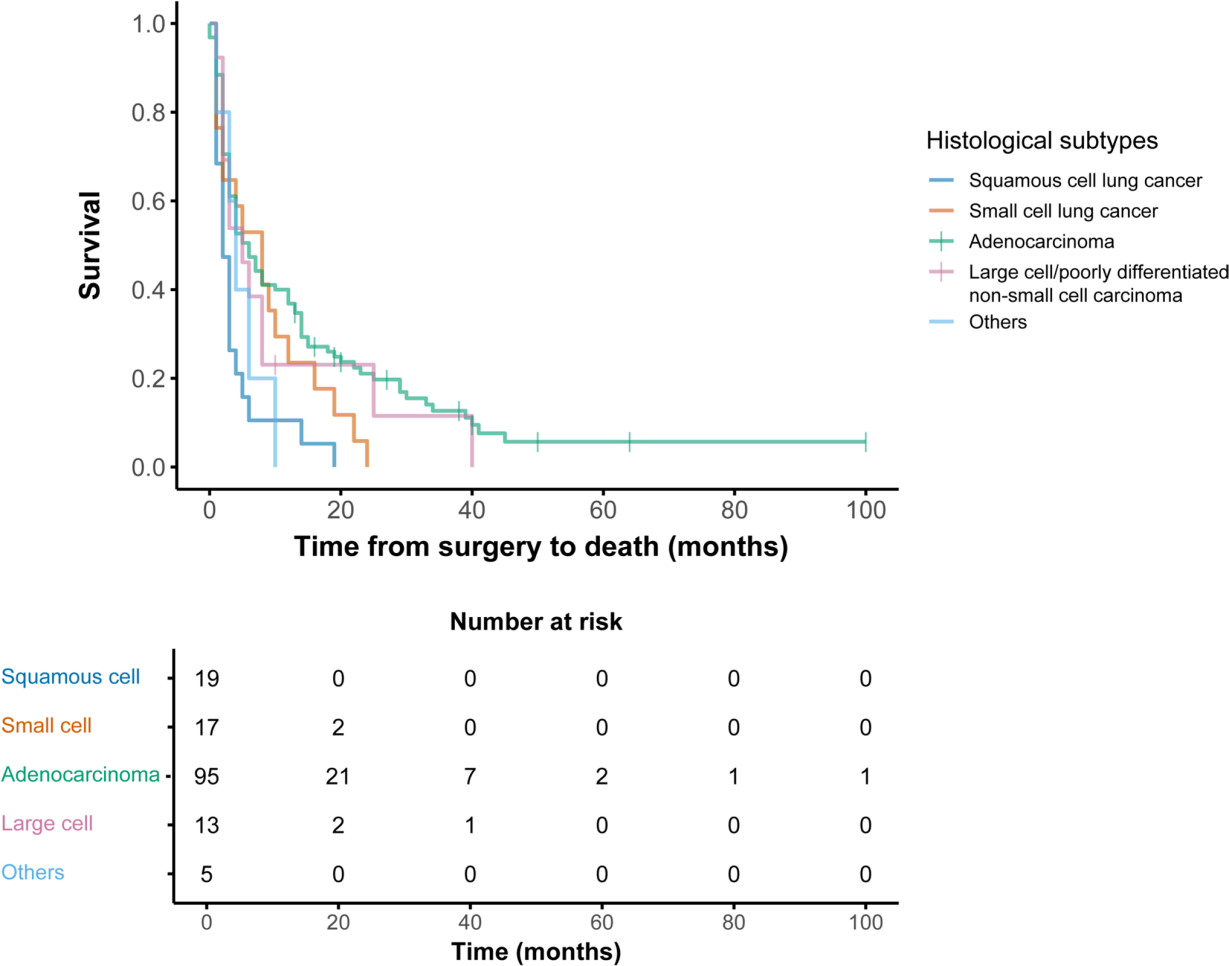
Survival after spinal surgery for lung cancer. Postoperative survival by histological subtype of lung cancer in patients undergoing spinal surgery. Kaplan–Meier curves demonstrate significant differences in overall survival between subtypes (*p* = 0.004). Median survival times were 6.0 months (95% CI 3.6–8.4) for adenocarcinoma (*n* = 95), 2.0 months (95% CI 0.9–3.1) for squamous cell carcinoma (*n* = 19), 8.0 months (95% CI 2.7–13.3) for small cell lung cancer (*n* = 17), 5.0 months (95% CI 1.5–8.5) for large cell/poorly differentiated non-small cell carcinoma (*n* = 13), and 4.0 months (95% CI 1.9–6.1) for tumors classified as “other” (*n* = 5). The “other” category included adenosquamous carcinoma (*n* = 1), pleomorphic/sarcomatoid carcinoma (*n* = 1), and unclassified cases (*n* = 3)

The median survival after spinal surgery for the entire cohort was 4 months (95% CI 2–6). Postoperative survival was significantly associated with histological subtype of lung cancer (*p* = 0.004) (Fig. 2). Patients with spinal metastasis as the IMM had a median survival of 6 months (95% CI 4–8), compared to 3 months (95% CI 2–4) in those with a previously established lung cancer diagnosis (*p* = 0.022). There were no differences in postoperative survival between national regions (*p* = 0.984) or in year of surgery. In the latter, data were stratified in two groups: spinal surgery before 2016 (*n* = 73) and spinal surgery after 2016 (*n* = 76) (*p* = 0.096).

**Fig. 3:**
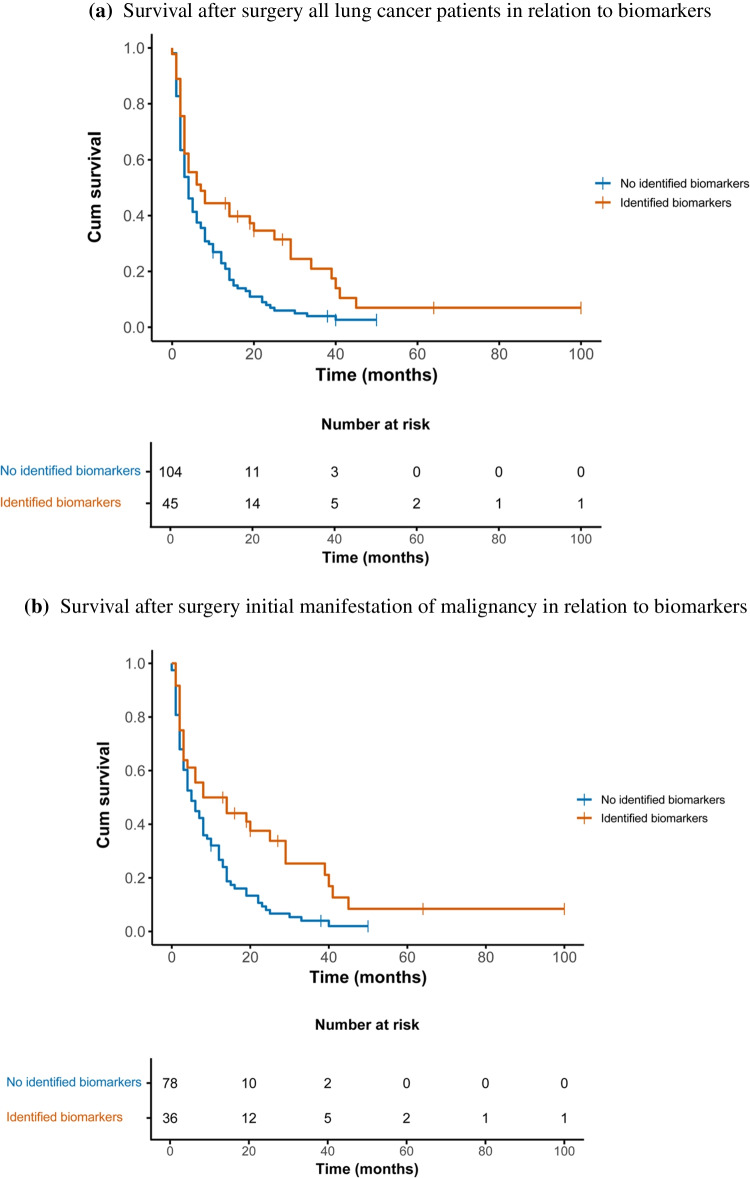
**A** Survival after surgery all lung cancer patients in relation to biomarkers. **b** Survival after surgery initial manifestation of malignancy in relation to biomarkers. **A** Survival after surgery in all patients with lung cancer and spinal metastases, stratified by presence of targetable biomarkers. Patients with identified biomarkers (*n* = 45) had a median survival of 7 months (95% CI 2–12), compared to 4 months (95% CI 3–5) in patients without identified biomarkers (*n* = 104) (*p* = 0.002). **B** Survival in the subgroup of patients with spinal metastases as the initial manifestation of lung cancer, stratified by biomarker status. Median survival was 8 months (95% CI 0–19) in patients with identified biomarkers (*n* = 36), and 5 months (95% CI 3–7) in patients without identified biomarkers (*n* = 78) (*p* = 0.003)

Predictive biomarkers were identified in 45 patients, who demonstrated a significantly longer median postoperative survival of 7 months (95% CI 2–12), compared to 4 months (95% CI 3–5) in patients without identifiable biomarkers (*n* = 104, *p* = 0.002). In the subgroup with spinal metastases as the IMM, the presence of predictive biomarkers was associated with improved postoperative survival: 8 months (95% CI 0–19) vs. 5 months (95% CI 3–7) (*p* = 0.003) (Fig. 3a–b). No survival difference related to biomarker status was observed in patients with known lung cancer after spinal surgery (*p* = 0.353).

**Fig. 4:**
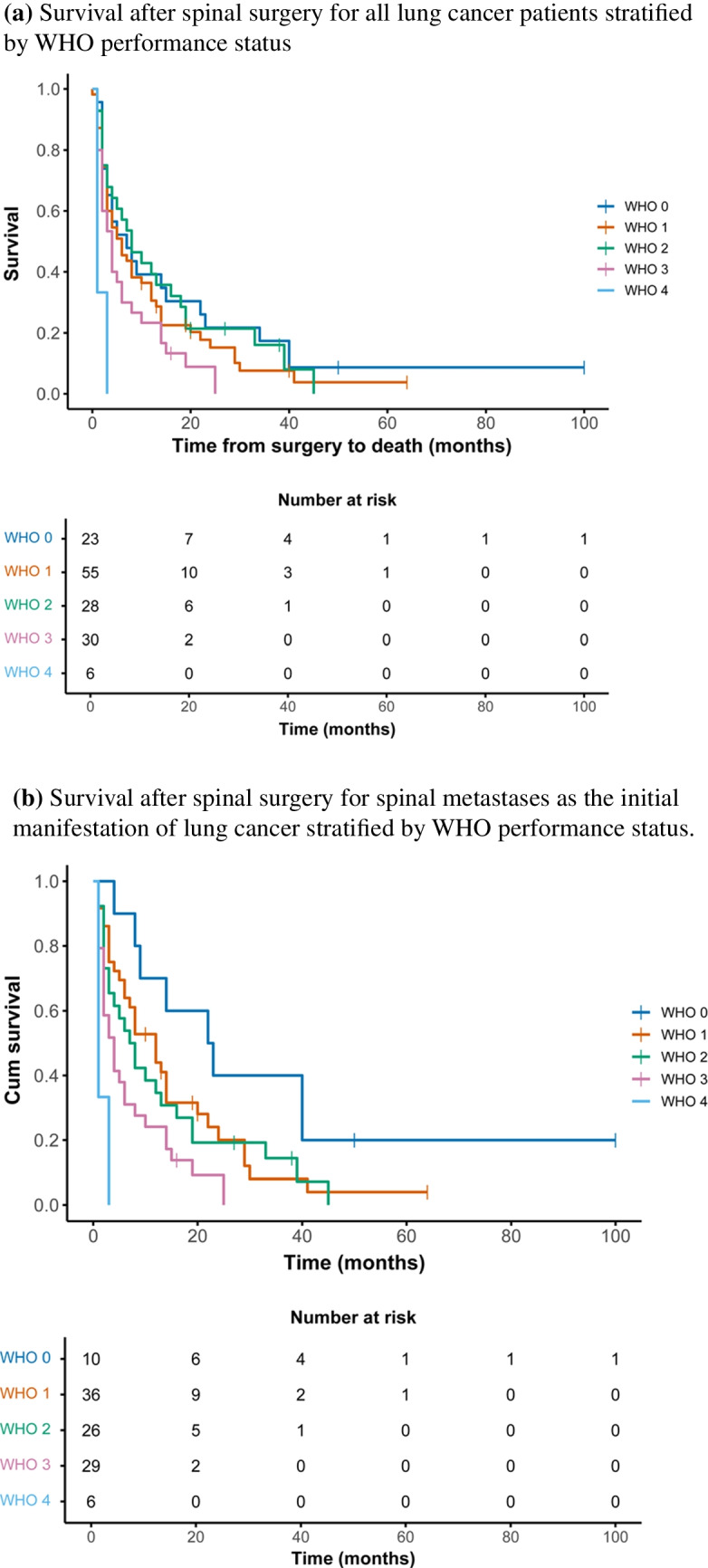
**a** Survival after spinal surgery for all lung cancer patients stratified by WHO performance status. **b** Survival after spinal surgery for spinal metastases as the initial manifestation of lung cancer stratified by WHO performance status. **A** Survival in the entire cohort of patients with lung cancer and spinal metastases undergoing surgery. Median survival was 7 months (95% CI 1–13) for patients with WHO performance status 0, and 1 month (95% CI 0–2) for patients with WHO 4 (*p* < 0.001). **B** Survival in the subgroup of patients with spinal metastases as the initial manifestation of lung cancer. Median survival was 22 months (95% CI 8–38) for WHO 0, 12 months (95% CI 7–16) for WHO 1, 7 months (95% CI 3–11) for WHO 2, 4 months (95% CI 2–6) for WHO 3, and 1 month (95% CI 0–1) for WHO 4 (*p* < 0.001)

Median postoperative survival was 8 months (95% CI 1–15) in ambulatory patients, compared to 3 months (95% CI 2–5) in non-ambulatory patients (*p* = 0.002). WHO performance status was also significantly associated with survival (*p* < 0.001). In patients with spinal metastasis as IMM, median survival ranged from 22 months (WHO 0) to 1 month (WHO 4) (*p* < 0.001) (Fig. 4a–b), while no significant survival difference by WHO status was observed in patients with known lung cancer (*p* = 0.251).

In multivariate Cox regression analysis restricted to patients with spinal metastases as the IMM, independent predictors of postoperative survival included WHO performance status, presence of a predictive biomarker, ambulation, and age at surgery. Specifically, higher WHO performance status was associated with an increased risk of death, with HR 2.94 (95% CI 1.14–7.77, *p* = 0.026) for WHO 3 and HR 6.58 (95% CI 1.83–23.72, *p* = 0.004) for WHO 4, compared to WHO 0. Patients harbouring a presence of a predictive biomarker had a reduced hazard (HR 0.52, 95% CI 0.31–0.86, *p* = 0.011). Non-ambulatory patients had increased hazard compared to ambulatory patients (HR 1.67, 95% CI 1.03–2.71, *p* = 0.037). Older age at surgery was associated with slightly increased hazard (HR 1.03 per year, 95% CI 1.00–1.06, *p* = 0.022). Sex was not significantly associated to postoperative survival (HR 1.05, 95% CI 0.64–1.71, *p* = 0.861). The results were consistent across both Model 1, which included only complete cases, and Model 2, which used multiple imputation (Table 3). Similarly, when stratifying the subgroup of patients who underwent spinal surgery as the IMM using criteria of primary lung cancer diagnosis within 30 or 60 days of spinal surgery the findings remained largely unchanged. The only exception was ambulation, which lost statistical significance in the Cox proportional hazards model when using the 30-day criterion (HR 1.64, 95% CI 0.97–2.79, *p* = 0.064).

**Table 3:** Variables associated with risk of death after spinal surgery for lung cancer as the initial manifestation of malignancy

Variable	HR	95% CI (Model 1)	*P*-value (Model 1)	HR	95% CI (Model 2)	*P*-value (Model 2)
	(Model 1)			(Model 2)		
WHO performance status			0.029			< 0.001
0 (Ref)	1.00	–	–	1.00	–	–
1	1.75	0.73–4.19	0.210	1.85	0.80–4.26	0.148
2	1.67	0.69–4.09	0.258	1.83	0.78–4.31	0.168
3	2.94	1.14–7.57	0.026	2.91	1.19–7.13	0.020
4	6.58	1.83–23.72	0.004	8.44	2.85–25.00	< 0.001
Predictive biomarker			0.011			0.003
Negative (Ref)	1.00	–	–	1.00	–	–
Positive	0.52	0.31–0.86	–	0.49	0.31–0.78	–
Ambulation			0.037			0.005
Ambulatory (Ref)	1.00	–	–	1.00	–	–
Non-ambulatory	1.67	1.03–2.71	–	1.82	1.2–2.77	–
Sex			0.861			0.781
Female (Ref)	1.00	–	–	1.00	–	–
Male	1.05	0.64–1.71	–	1.06	0.70–1.61	–
Age at surgery	1.03	1.00–1.06	0.022	1.03	1.0–1.1	0.014

Cox proportional hazards analyses comparing complete-case data (Model 1) and multiply imputed data (Model 2). Model 1 presents hazard ratios (HR), 95% confidence intervals (CI), and *p*-values based on complete observations, whereas Model 2 provides corresponding estimates after multiple imputation of missing data

### Patient reported outcomes

Patient-reported quality-of-life data, including EQ-5D, were assessed at 6 weeks after surgery. Fifty-one out of 62 patients (84%) reported decreased pain after surgery and 30 out of 62 patients (49%) reported better motor function (Supplementary Tables 1 and 2).

## Discussion

In this large national cohort study, we found that the overall postoperative survival for patients undergoing surgery for spinal metastases deriving from lung cancer was short. Most patients (77%) presented with spinal metastases as the initial manifestation of lung cancer (IMM). In this subgroup, key clinical and molecular predictors including performance level, ambulatory status, and specific targetable biomarkers, were associated with improved postoperative survival.

Lung cancer is traditionally associated with poor prognosis, but recent advances in targeted therapies have changed the outlook for selected molecular subtypes with targetable biomarkers [10]. Recently Takahashi et al. [20] reported in a prospective cohort study of 74 patients that biomarker profiles were associated with longer survival after surgery for lung cancer patients with spinal metastases. Their cohort included patients with a known lung cancer diagnosis, some of whom had received prior oncological treatment. Our findings support that biomarkers and the advancements target therapy is important for proper estimation of survival after spinal surgery by identifying a subgroup of treatment-naïve patients with molecular alterations who experience improved survival following surgery. In particular, the presence of biomarkers in patients with IMM had significantly longer median survival compared to those without biomarkers or not tested.

Even though these patients were already selected for surgery, postoperative survival remained generally poor, underscoring the limited life expectancy in this population. Following the randomized controlled trial by Patchell et al. [17], surgical decompression rapidly became a cornerstone in the management of metastatic spinal disease. However, recent findings by Migliorini et al. [14], showing that single-fraction 8 Gy radiotherapy yields effective pain relief without negatively affecting survival, highlights the value of radiotherapy as a palliative modality and emphasize the need for rigorous patient selection for surgical intervention. In patients with limited expected survival, radiotherapy alone may represent the most appropriate primary treatment, whereas surgery should be reserved for those with a reasonable prognosis, with the potential benefits of operative management.

Previous studies have emphasized the prognostic role of mutations in metastatic lung cancer. In a systematic review by Batista et al. [1], patients with spinal metastases, expression of EGFR mutations had significant better survival. Dongzono et al. [6] reported a median survival of 21.4 months in patients with EGFR mutations receiving targeted therapy, although only 15% underwent surgery. Zhai et al. [27] observed similar patterns in a surgical cohort, with a marked survival benefit in those receiving targeted therapies. Interestingly, patients who initiate target therapy following treatment for spinal metastases have demonstrated improved survival compared to those who were already receiving target therapy prior to spinal surgery [4]. Similar, while the presence of predictive biomarkers was significantly associated with survival in the IMM group, no such association was found for patients with previously known lung cancer. This suggests that patients who develop spinal metastases later in the disease progression, despite previous systemic therapy, may have little to gain from surgery, even in the presence of mutations. In contrast, patients newly diagnosed with lung cancer with targetable mutations had longer postoperative survival and these patients may thus benefit from surgery before proceeding to systemic treatment, provided their performance status and neurological function are preserved.

Several prognostic scoring systems have been developed to aid clinicians in estimating survival and guiding surgical decision-making in patients with spinal metastases. However, these tools have limitations in accuracy and may not adequately account for advances in oncological therapies, particularly for patients with unknown primary tumours at the time of spinal surgery or emerging molecular profiles [2, 7, 15, 22, 23] since these patients are in a treatment naïve stage at the time of spinal metastases.

Our study contributes to the existing literature by focusing specifically on patients in whom spinal metastasis was the initial manifestation of malignancy, highlighting the opportunity for timely diagnosis and therapeutic intervention at a potentially earlier stage of disease. Moreover, by characterizing this distinct patient group, our findings may support more accurate estimation of postoperative survival and thereby inform individualized treatment planning.

Ambulatory status and WHO performance status were associated with postoperative survival, consistent with existing literature [11, 15, 17]. Interestingly, lung cancer patients with spinal metastases as the initial manifestation and high-performance status had increased median survival after surgery. Poor performance status (WHO 3–4) and non-ambulatory (Frankel A-C) patients had significantly reduced survival, emphasizing the importance of functional assessment when considering surgical intervention. These clinical indicators remain critical in decision-making, particularly when molecular testing is unavailable. A significant clinical challenge persists, when metastatic spinal cord compression with rapidly progressive neurological deficits requiring urgent surgery is the first manifestation of malignancy. This scenario limits the extent of preoperative diagnostic workup and, together with referral-related selection bias, likely contributes to a higher proportion of patients undergoing surgical treatment.

From a surgical perspective, our findings must be interpreted in the context of real-world decision-making, where molecular and histopathological data are often unavailable at the time of surgery. In most cases, decisions are therefore based on neurological status, radiological findings, and performance status.

Our results suggest that spinal metastasis as the initial manifestation of malignancy may represent a clinically relevant surrogate of disease stage. Patients with treatment-naïve lung cancer, despite systemic spread, may differ prognostically from those with previously treated disease, where progression may reflect more advanced or therapy-resistant tumour biology.

While biomarkers were associated with survival, these are rarely available in the acute setting. In contrast, the recognition of previously untreated disease is immediately accessible and may support a lower threshold for surgical intervention in selected patients, even in tumour entities traditionally associated with poor prognosis such as lung cancer.

The quality of life measures in this study should be interpreted with caution, as the substantial amount of missing data limits the strenght of any conclusion and renders the results exploratory. The available data suggest that most patients experienced improvement at 6 weeks after surgery. Previous studies, such as Tang et al. [21], have reported improved quality-of-life outcomes in lung cancer patients with bone metastases who underwent surgical treatment compared with those managed non-surgical. Given the limited evidence on quality of life after spinal surgery in lung cancer patients, our findings highlight the need for further research to better inform patient-centred care and expectation management.

### Strength and limitation

This study is based on a large, nationally representative cohort with high data completeness, derived from two well-established quality registries in Sweden. The integration of detailed surgical and oncological data, including molecular biomarkers and functional status, enabled a comprehensive analysis of a clinically important and previously underexplored patient group: those presenting with spinal metastases as the initial manifestation of lung cancer. This approach allowed identification of key prognostic factors associated with postoperative survival, offering valuable insights into patient selection and the timing of surgical intervention. A potential limitation relates to the Swespine registry. Although the registry demonstrates an overall completeness rate exceeding 85%, the completeness specific to metastatic spine surgery has not been reported separatly, and may therefore be lower given the urgent nature of these procedures, introducing a risk of underreporting and selection bias.

To accurately capture these patients, we applied a 90-day diagnostic window following surgery, accounting for real-world delays in diagnostic workup, molecular profiling, and referral pathways across specialties. The intention was to identify patients who were clinically early in their metastatic course and therefore more likely to remain candidates for adjuvant or early systemic therapy. We performed sensitivity analyses using alternative thresholds of 30 and 60 days. These analyses produced similar hazard estimates, indicating that our main findings are robust to reasonable variation in the definition of early presentation.

Another potential limitation is the potential for immortal time bias, as postoperative diagnostic confirmation may require patients to survive beyond the date of surgery, the short diagnostic intervals in most patients and the consistency of our sensitivity analyses suggest that the impact of this bias is limited. Biomarker testing, neurological assessments, and quality-of-life data were incomplete in a proportion of patients, which may limit the generalizability of certain findings. Another limitation is that EGFR, ALK, ROS1, BRAF, RET, and PD-L1 were analyzed together as “predictive biomarkers,” despite representing biologically distinct categories with different therapeutic implications. Grouping these markers may therefore obscure subtype-specific prognostic differences. In addition, the absence of a non-surgical control group limits our ability to determine the direct survival benefit of surgery. Finally, registry data did not include sufficient detail on the timing, sequence, or intensity of postoperative systemic therapies, preventing adjustment for treatment initiation dates, which may act as an important confounder. The subgroup of patients with a previously known lung cancer diagnosis was relatively small (*n* = 35) compared with those without a known diagnosis at the time of surgery (*n* = 114), which may limit statistical power and the generalisability of subgroup-specific findings.

Despite these limitations, the study underscores the clinical relevance of molecular and functional parameters in guiding surgical decisions in this complex, treatment-naïve patient population.

## Conclusion

Among patients undergoing surgery for spinal metastases from lung cancer, those presenting with spinal metastasis as the initial manifestation of malignancy represent a distinct and clinically important subgroup. In these patients, presence of biomarkers, ambulatory function, and performance status were significantly associated with longer survival, suggesting that surgery may offer meaningful benefit when integrated into a multimodal treatment plan. Future strategies should incorporate analysis of biomarkers to further improve surgical decision-making among patients with treatment-naïve lung cancer presenting with spinal metastases.

## Supplementary Information

Below is the link to the electronic supplementary material.

ESM 1 Supplementary Material 1 (DOCX 16.6 KB)

ESM 2 Supplementary Material 2 (DOCX 16.1 KB)

## Data Availability

No datasets were generated or analysed during the current study.

## Declarations

### Reporting guidelines

The reporting of this observational study complies with the STROBE (Strengthening of Reporting of Observational Studies in Epidemiology) checklist.
