# Supplementary material for: Treatment-naïve lung cancer presenting with spinal metastases: a national study of survival, surgery, and the role of predictive biomarkers
Source: Acta Neurochir (Wien). 2026 May 19;168(1):111. doi: 10.1007/s00701-026-06914-3 (PMC13190795; doi:10.1007/s00701-026-06914-3)
Supplement: Supplementary file 1 — Supplementary Material 1 (DOCX 16.6 KB) [file 701_2026_6914_MOESM1_ESM.docx]

**Supplementary Table 1:** Patient reported outcomes from Swespine, 6 week after surgery for spinal metastases from lung cancer (n=62).

| **Pain (Change from baseline)** | **Number of patients (%)** |
| --- | --- |
| No pain prior to intervention | 4 (2.7) |
| Reduced pain | 51 (34.2) |
| Unchanged pain | 5 (3.4) |
| Increased pain | 2 (1.3) |
| Missing data | 87 (58.4) |
| **Motor function** |  |
| Improved motor function | 30 (20.1) |
| Unchanged motor function | 13 (8.7) |
| Deteriorated motor function | 19 (12.8) |
| Missing data | 87 (58.4) |
| **Ambulatory function** |  |
| Full ambulatory capacity | 12 (8.1) |
| Ambulatory with assistive device(s) | 16 (10.7) |
| Ambulation with cane support | 3 (2.0) |
| Ability to stand but predominantly wheelchair-dependent | 15 (10.1) |
| Non-ambulatory (unable to stand or walk) | 15 (10.1) |
| Missing data | 88 (59.1) |
| **Level of Care** |  |
| Independent living | 54 (36.2) |
| Long-term intitutional care | 3 (2.0) |
| Other level of care | 5 (3.4) |
| Missing data | 87 (58.4) |
