# Supplementary material for: Treatment-naïve lung cancer presenting with spinal metastases: a national study of survival, surgery, and the role of predictive biomarkers
Source: Acta Neurochir (Wien). 2026 May 19;168(1):111. doi: 10.1007/s00701-026-06914-3 (PMC13190795; doi:10.1007/s00701-026-06914-3)
Supplement: Supplementary file 2 — Supplementary Material 2 (DOCX 16.1 KB) [file 701_2026_6914_MOESM2_ESM.docx]

**Supplementary Table 2:** Patient reported quality-of-life outcomes (EQ-5D) from Swespine, 6 week after surgery for spinal metastases from lung cancer (n=60).

| **Pain** | **Number of patients (%)** |
| --- | --- |
| No pain or discomfort | 2 (1.3) |
| Moderate pain or discomfort | 49 (32.9) |
| Severe pain or discomfort | 9 (6.0) |
| Missing data | 89 (59.7) |
| **Anexiety** |  |
| No anexiety | 25 (16.8) |
| Moderate anxiety | 31 (20.8) |
| Severe anexiety | 4 (2.7) |
| Missing | 89 (59.7) |
| **Usual daily Activities** |  |
| No problems performing usual activities | 10 (6.7) |
| Some problems performing usual activities | 24 (16.1) |
| Unable to perform usual activities | 26 (17.4) |
| Missing data | 89 (59.7) |
| **Self-Care** |  |
| No problem with self-care | 27 (18.1) |
| Some problems with self-care | 23 (15.4) |
| Unable to wash or dress oneself | 10 (6.7) |
| Missing data | 89 (59.7) |
| **Mobility** |  |
| Able to walk without difficulty | 10 (6.7) |
| Able to walk with some difficulty | 35 (23.5) |
| Confined to bed | 15 (10.1) |
| Missing data | 89 (59.7) |
